# Case Report: A case of hepatic encephalopathy secondary to a spontaneous splenorenal shunt

**DOI:** 10.3389/fsurg.2025.1693996

**Published:** 2026-01-21

**Authors:** Yulong An, Chao Deng, Chong Wen, Jinli Liu, Yongqiang Zhu, Kai Chen, Hao Luo

**Affiliations:** 1 Department of Hepatobiliary Surgery, The Affiliated Hospital of Southwest Medical University, Luzhou, China; 2 Department of General Surgery, The General Hospital of Western Theater Command, Chengdu, China; 3 Department of Hepatobiliary Surgery, Fokind Hospital, Tibet University, Lhasa, China

**Keywords:** hepatic encephalopathy, spontaneous splenorenal shunt, hypersplenism, cirrhosis, spontaneous portosystemic shunt

## Abstract

This report presents a case of hepatic encephalopathy (HE) induced by a spontaneous splenorenal shunt (SSRS). A 73-year-old male patient was admitted to our medical facility due to loss of consciousness. Laboratory analyses revealed elevated blood ammonia levels and varying degrees of reduction in erythrocyte, leucocyte, and platelet levels. Portal vein imaging utilizing 320-slice CT demonstrated enlargement of the portal and splenic veins, splenomegaly, multiple varicose veins at the splenic hilum, and local protrusion of the left renal vein. An initial diagnosis of HE with SSRS and hypersplenism was established. A multi-disciplinary treatment approach was implemented, incorporating a patient–doctor collaborative decision-making model. Two treatment options were presented to the patient, who opted for surgical intervention over interventional treatment. Subsequently, a combined splenectomy and splenorenal shunt vessel ligation procedure was performed. Postoperatively, the patient's condition exhibited significant improvement compared to his pre-operative state, with no recurrence of HE observed. This article reports a case of recurrent hepatic encephalopathy and severe hypersplenism related to SSRS, which was successfully treated by combined splenectomy and vascular disconnection.

## Introduction

A spontaneous splenorenal shunt (SSRS) is characterized by the proliferation and enlargement of abnormal blood vessels connecting the splenic vein to the renal vein. This vascular anomaly is predominantly located in the left subphrenic region, originating from the splenic portal vein and terminating in the left renal vein. SSRSs account for 30.3% of atypical collateral circulation cases (1). The presence of an SSRS can significantly alleviate portal vein pressure, mitigate symptoms associated with portal hypertension, and reduce the incidence of esophageal variceal (EV) rupture bleeding and ascites formation (2). However, an SSRS also has significant drawbacks. A substantial portion of blood flow bypasses the liver's detoxification processes, allowing toxins to enter the systemic circulation directly. This bypass mechanism can adversely affect the chronic progression of cirrhosis and potentially induce or exacerbate hepatic encephalopathy (HE) and other related complications.

HE is defined as a neuropsychiatric syndrome that manifests due to severe hepatic dysfunction or abnormal portal veno-systemic shunting (3). While HE is typically associated with severe liver cirrhosis or portosystemic shunting, its occurrence in patients with a spontaneous portosystemic shunt (SPSS) is relatively uncommon.

Although interventional embolization [balloon-occluded retrograde transvenous obliteration (BRTO), coil-assisted retrograde transvenous obliteration (CARTO), and vascular plug-assisted retrograde transvenous obliteration (PARTO)] manages most cases of SSRS-HE with 60%–100% short-term neurological success (4, 5), it is associated with significant complications. These include aggravated portal hypertension—evidenced by a 27.4% rebleeding rate in one study—and new collateral formation, which can necessitate additional surgical procedures (6). Crucially, how to manage the particular clinical scenario of patients with an SSRS who have severe hypersplenism requiring splenectomy yet refuse interventional embolization remains unreported in indexed studies.

This case demonstrates that open splenectomy with SSRS ligation is a viable and effective alternative for patients with an SSRS who refuse interventional therapy. The procedure achieved immediate resolution of the patient’s hepatic encephalopathy, corrected his life-threatening pancytopenia, and resulted in permanent shunt occlusion on postoperative CT—thereby obviating the risk of endovascular recanalization. It thus represents a viable surgical alternative for patients with an SSRS who are unsuitable for or unwilling to undergo interventional procedures.

## Case description

**Figure 1:**
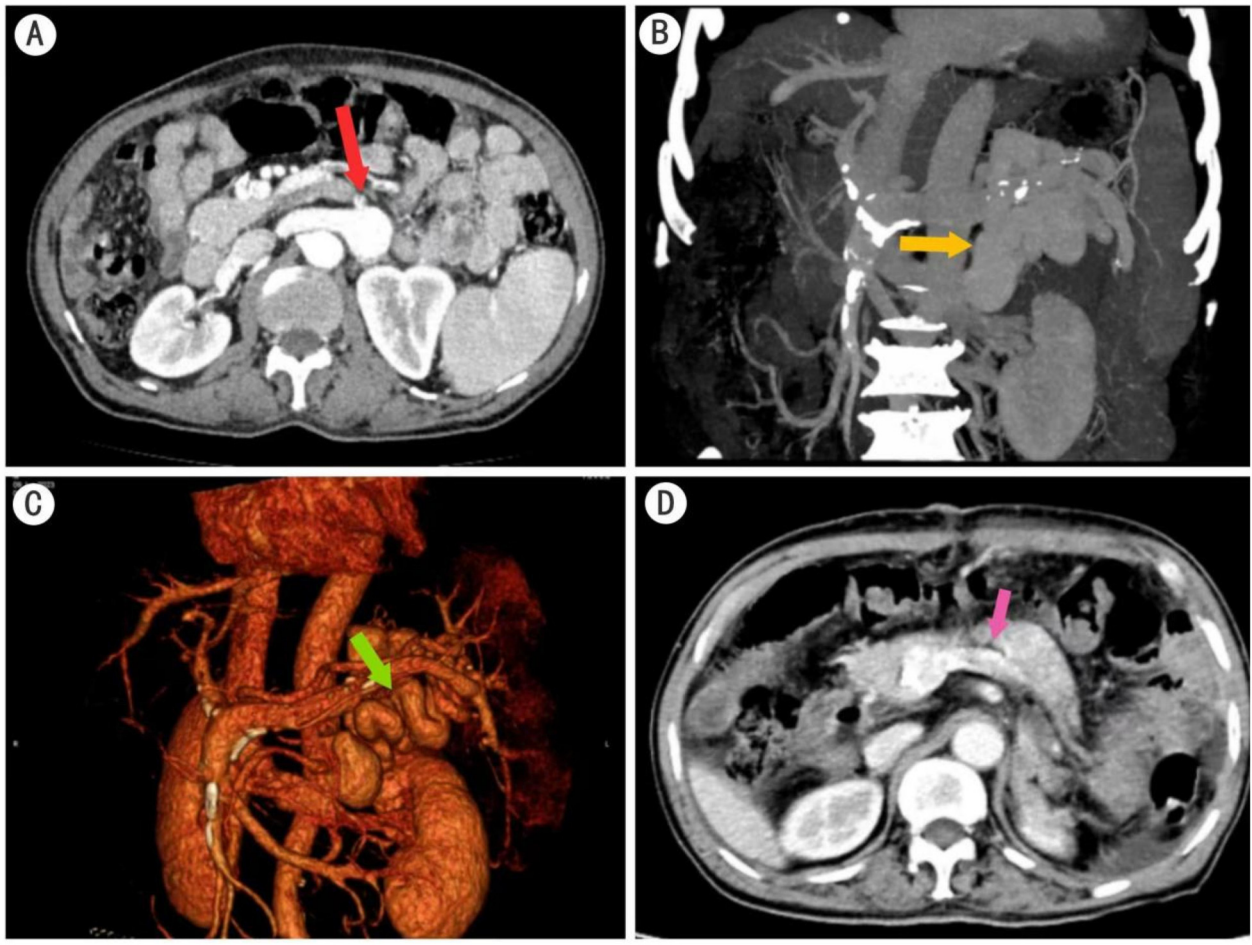
320-slice CT portal vein imaging. **(A)** The coronal view demonstrates enlargement of the splenorenal shunt. **(B,C)** Sagittal views reveal the thickened and tortuous splenorenal shunt. **(D)** Postoperative image shows the absence of the spleen and narrowing of the previously dilated portal vein with complete obliteration of the splenorenal shunt.

A 73-year-old male patient was admitted to our medical facility for the third time due to loss of consciousness. Comprehensive laboratory tests were conducted, revealing elevated blood ammonia and bilirubin levels, decreased albumin concentration, and prolonged prothrombin time. Hematological findings demonstrated pancytopenia, consistent with the laboratory diagnosis of hypersplenism. However, the patient remained clinically asymptomatic, with no reports of epistaxis, purpura, abdominal distension, or pain. Abdominal ultrasonography yielded a sonographic profile consistent with cirrhosis. Neuroimaging studies, including head CT and MRI scans, and cardiovascular assessments, such as an electrocardiogram and echocardiogram, did not reveal any significant abnormalities. A thorough neurological examination conducted by a specialist failed to identify any specific pathological signs. Consequently, encephalopathies of cerebral or cardiac origin were excluded from the differential diagnosis. Gastroscopic evaluation showed no evidence of varicose veins, and the gastric fundus exhibited normal mucosal integrity. To elucidate the etiology of the HE, 320-row CT portal vein angiography was performed (Figure 1). The imaging study revealed a portal vein diameter of approximately 1.5 cm, with the splenic vein exhibiting marked tortuosity and dilation, measuring approximately 1.4 cm in width. Splenomegaly was observed, accompanied by multiple varicose veins at the splenic hilum. A localized cystic protrusion and thickening of the left renal vein were noted, measuring approximately 2.5 cm × 2.2 cm × 3.8 cm. The radiological findings were indicative of cirrhosis, splenomegaly, and portal hypertension with associated collateral circulation development.

The patient presented with alcohol-related cirrhosis secondary to chronic ethanol intake exceeding 100 g/day for more than 20 years, alongside a history of recurrent ascites. Baseline liver function was severely compromised, indicated by a Child–Pugh score of 11 (class C) and a Model for End-Stage Liver Disease score of 19. The index admission in June 2023 was triggered by a deterioration to West Haven grade III hepatic encephalopathy, representing the third episode within a 6-month period since January 2023, with two preceding grade-II events.

**Figure 2:**
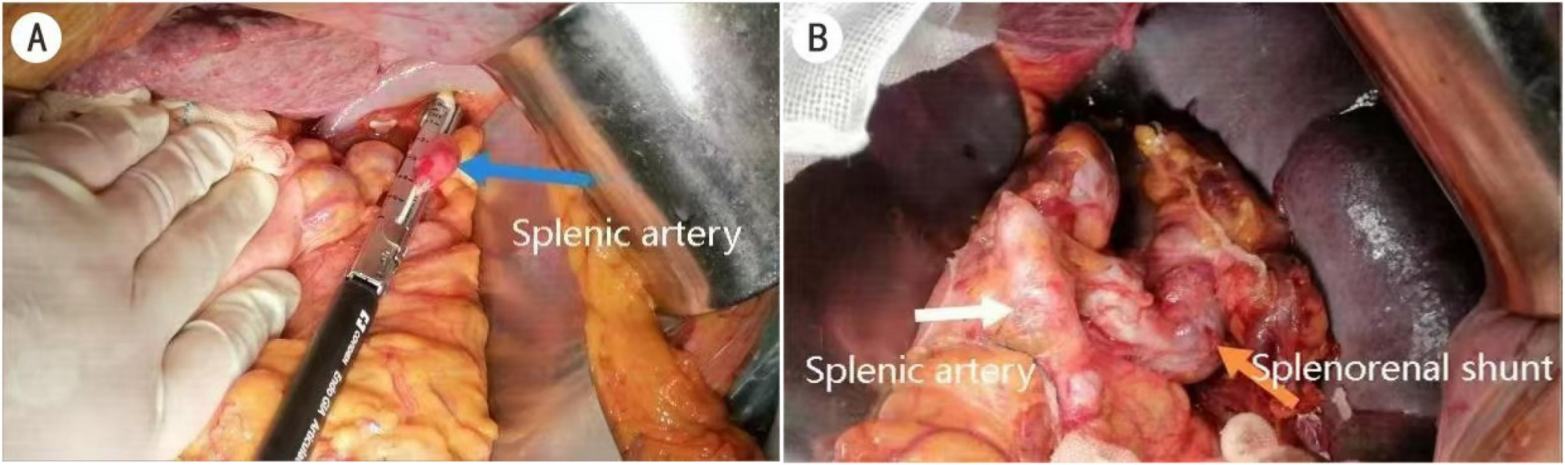
Surgical procedure. **(A)** Ligation of the splenic artery. **(B)** Exposure of the splenorenal shunt vessel branch.

Despite the patient's history of cirrhosis, no precipitating factors for liver failure, such as gastrointestinal bleeding or infection, were identified. Pancytopenia [red blood cell (RBC): 3.06 × 10^12^/L; platelet (PLT): 70 × 10⁹/L; white blood cell (WBC): 2.89 × 10⁹/L] with grade III HE and a large SSRS constituted definitive indications for combined splenectomy and SSRS disconnection. On pre-operative reassessment, the patient remained at West Haven grade III with a Child–Pugh score of 10 (class C), showing no significant improvement in liver function. Consequently, splenectomy and splenorenal shunt vessel ligation were performed. Intraoperative findings revealed an enlarged spleen measuring 16 cm, with notably thickened and tortuous splenic hilar vessels. The markedly enlarged and tortuous splenic artery (Figure 2A) was isolated, ligated, and transected. Upon dissection of the splenic vein towards its lower pole, a tortuous vessel (Figure 2B) was observed coursing medially, inferiorly, and posteriorly. Corroborating with the pre-operative CT findings, this vessel was identified as the splenic vein shunting to the left renal vein and was subsequently ligated and divided. The main splenic vein, upper splenic pole branch, and a small portion of the pancreatic tail were ligated and transected, followed by complete splenectomy.

### Postoperatively

The patient demonstrated marked symptomatic and laboratory improvement postoperatively (Table 1). Drain output was serosanguineous (approximately 150 mL) on postoperative day (POD) 1 and decreased to 30–50 mL of serous fluid by POD 5. The recovery was uneventful, with no evidence of hemorrhage, pancreatic fistula, or chyle leak. Mild postoperative ascites resolved with conservative management (diuretics and albumin), and there was no occurrence of spontaneous bacterial peritonitis.

**Table 1:** Laboratory values before and after surgery.

Parameter	Pre-op	Post-op 1 m	Post-op 12 m	Reference range
Ammonia (μmol/L)	105.1	22.4	9.1	18–72
WBC (×10^9^/L)	2.89	3.26	3.32	3.5–9.5
PLT (×10⁹/L)	70	139	213	125–350
Albumin (g/L)	32.2	37.3	41.9	40–55
International normalized ratio	1.50	1.23	1.12	0.8–1.2
Total bilirubin (μmol/L)	39.4	21.2	20.9	5–28
RBC (×10^12^/L)	3.06	3.26	3.42	4.3–5.8

Follow-up CT imaging (Figure 1D) revealed post-splenectomy changes, including two retained drainage tubes in the surgical field, blurring of the surrounding fat planes, and thickening of the left renal anterior fascia. Radiological features consistent with cirrhosis and portal hypertension with collateral circulation formation were also noted. At the 2-year follow-up, the patient remained free from recurrence of HE. Moreover, substantial improvements were observed in blood ammonia levels, liver function parameters, and hematological indices.

## Discussion

The patient was initially admitted to the neurology department for differential diagnosis of unconsciousness. Through comprehensive imaging examinations, cerebral hemorrhage, infarction, brain tumors, and other brain-derived encephalopathies were excluded. Cardiogenic encephalopathy was ruled out by an electrocardiogram and color Doppler ultrasound. Based on the analysis of altered consciousness symptoms and elevated plasma ammonia levels, the clinical presentation was consistent with HE. Hematological analysis revealed varying degrees of reduction in erythrocytes, leucocytes, and platelets, indicative of hypersplenism. In conjunction with abdominal CT findings and the patient's medical history obtained from the general practitioner, the primary diagnosis was established as “HE with SSRS.”

Previous studies have reported the relative incidence of SSRSs in patients with portal hypertension to be 7% when assessed by portal venography and 10.7% in cirrhotic patients when evaluated by transabdominal ultrasound (7).

The pathophysiology of SSRS remains incompletely understood. Tafel and Lejars postulate that these pre-existing venules represent anastomoses between the renal fat cystic vein and spleen-dependent venules located in the retropancreatic papillary tissue. The veins in the perirenal fat form an extensive mesh network, termed “repulsion,” through a vertical anastomosis system connecting the suprarenal current (linked to the upper renal vein) and the subrenal current (connected to the renal vein) (8). Under conditions of increased circulatory pressure, such as portal hypertension, this network undergoes significant development, with vessels increasing in caliber and evolving into prominent veins (9).

An SSRS is typically located within the splenorenal ligaments, representing a vascular pathway that was occluded or partially occluded during embryonic development. Under conditions of portal hypertension, these dormant vessels may recanalize. Concurrently, vasoactive substances such as nitric oxide and vascular endothelial growth factor enhance visceral circulation blood flow, further promoting vascular reopening (10, 11).

Diagnosis of an SSRS primarily relies on advanced imaging techniques. Enhanced CT and MRI offer improved spatial resolution, cross-sectional visualization, and comprehensive shunt vein imaging for SSRS detection. These modalities facilitate accurate measurement of shunt vessel diameters and delineation of spatial relationships between shunt vessels and portal veins. Furthermore, detailed analysis of portal vein thrombi, cavernous transformation, and other vascular anomalies can be conducted (12).

The primary treatment modality for SSRS-induced recurrent HE involves vascular interventional embolization techniques. These procedures include BRTO, CARTO, and PARTO (13). While conventional therapies aimed at ammonia reduction and control of predisposing factors can mitigate the frequency and severity of HE episodes, interventional radiology is indicated for persistent HE attacks. Research demonstrates that occlusion of a large SSRS results in nearly 100% short-term improvement in HE symptoms (4). Within 2 years post-intervention, approximately 60% of patients experience complete resolution of HE (5). Additionally, percutaneous transhepatic variceal embolization (PTVE) has emerged as an effective treatment for portal shunt-induced HE, specifically targeting collateral circulations such as splenorenal shunts.

Following interventional embolization of an SSRS, a reduction in shunt blood flow is observed, accompanied by improved liver function. However, the resultant increase in portal pressure warrants careful monitoring. Some patients may develop new-onset ascites or experience an exacerbation of pre-existing ascites following SSRS occlusion. The risk of EV bleeding necessitates endoscopic surveillance. Therefore, a comprehensive risk-benefit analysis should be conducted prior to SSRS occlusion for HE management. Early detection and intervention in HE-related SSRS cases are crucial to mitigate portal hypertension-associated adverse events. Caution is advised when considering vascular occlusion in patients with a history of EV bleeding, extensive portal thrombosis, or significant ascites (14). Intraoperative assessment of portal pressure, careful balancing of portal pressure–shunt relationships, and selective partial disconnection when necessary are recommended strategies to minimize postoperative complications. The optimal disconnection site should be meticulously selected. In high-risk patients with low portal pressure gradients, reduced portal blood flow to the liver, or exceptionally large SSRS diameters, the potential benefits of intervention may outweigh the associated risks.

The selection of interventional embolization or PTVE may exacerbate portal hypertension and splenomegaly. These limitations of interventional therapy underscore the need for alternative strategies, particularly in patients with severe hypersplenism. Previous studies have demonstrated the efficacy of splenectomy in ameliorating portal hypertension and improving liver function in patients with pancytopenia (15, 16). Nomura et al. observed a reduction in liver fibrosis area post-splenectomy in cirrhotic patients, accompanied by an increased ratio of CD8+ cells in peripheral blood. These findings suggest that splenectomy in hepatitis patients can mitigate liver fibrosis, induce beneficial immunological changes, and potentially reduce carcinogenesis risk (17). Moreover, corrective surgery involving the disconnection of splenorenal communicating vessels ensures hepatic clearance of blood ammonia, potentially offering a comprehensive solution for HE.

In the present case, a multi-disciplinary treatment approach was adopted, incorporating a patient–doctor joint decision-making model. Following collaborative consultations involving endoscopy, interventional radiology, anesthesiology, and other relevant departments, two treatment options were presented to the patient. The patient declined interventional treatment in favor of surgical intervention. Consequently, a combined splenectomy and splenorenal shunt vessel ligation procedure was performed.

Surgical splenectomy with SSRS ligation offers several distinct advantages over interventional embolization. First, it provides definitive, permanent shunt occlusion without the risk of recanalization inherent to endovascular techniques. Second, concomitant splenectomy simultaneously resolved the life-threatening hypersplenism, with the patient’s platelet count increasing from 70 to 213 × 10⁹/L at 12 months—a benefit that interventional approaches cannot provide. Third, our patient achieved a rapid neurological recovery (his ammonia level decreased from 105.1 to 22.4 μmol/L within 1 month) without precipitating portal hypertension complications such as variceal rebleeding or refractory ascites, which occur at a relatively high rate in patients post-embolization (6). While minimally invasive techniques remain the first-line treatment for the majority of patients with SSRS-HE, this case validates open surgical disconnection as a safe and effective alternative for those with severe hypersplenism or who decline interventional therapy, offering dual therapeutic benefits through a single intervention.

A principal limitation of this single-case study is the unavailability of intraoperative portal pressure measurements, which constrains our ability to quantify the hemodynamic consequences of shunt disconnection. Long-term radiological surveillance beyond 5 years is needed to assess for potential *de novo* collateral formation. Prospective registries or multicenter collaborations are warranted to validate surgical outcomes in this rare patient subset.

In conclusion, for patients with SSRS-associated HE and severe hypersplenism who refuse interventional therapy, this case demonstrates that combined splenectomy and SSRS ligation is a safe and effective surgical alternative. Prospective multicenter registries are needed to validate long-term outcomes and quantify hemodynamic changes in this rare patient subset.

## Data Availability

The original contributions presented in the study are included in the article/Supplementary Material, further inquiries can be directed to the corresponding author.
